# Implementation of buprenorphine services in NYC syringe services programs: a qualitative process evaluation

**DOI:** 10.1186/s12954-022-00654-0

**Published:** 2022-07-10

**Authors:** Andrea Jakubowski, Caroline Rath, Alex Harocopos, Monique Wright, Alice Welch, Jessica Kattan, Czarina Navos Behrends, Teresa Lopez-Castro, Aaron D. Fox

**Affiliations:** 1 Division of General Internal Medicine, Department of Internal Medicine, Albert Einstein College of Medicine/Montefiore Medical Center, 3300 Kossuth Avenue, Bronx, NY 10467 USA; 2 Bureau of Alcohol, Drug Use, Care, Prevention and Treatment, New York City Department of Health and Mental Hygiene, 42-09 28th Street Queens, Long Island City, NY 11101 USA; 3 Department of Population Health Sciences, Weill Cornell Medical College, 402 E. 67th St, New York, NY 10065 USA; 4 Department of Psychology, The City College of New York, 160 Convent Avenue, New York, NY 10031 USA

**Keywords:** Syringe services programs, Low-threshold buprenorphine treatment, Opioid use disorder

## Abstract

**Background:**

Syringe services programs (SSPs) hold promise for providing buprenorphine treatment access to people with opioid use disorder (OUD) who are reluctant to seek care elsewhere. In 2017, the New York City Department of Health and Mental Hygiene (DOHMH) provided funding and technical assistance to nine SSPs to develop “low-threshold” buprenorphine services as part of a multipronged initiative to lower opioid-related overdose rates. The aim of this study was to identify barriers to and facilitators of implementing SSP-based buprenorphine services.

**Methods:**

We conducted 26 semi-structured qualitative interviews from April 2019 to November 2019 at eight SSPs in NYC that received funding and technical assistance from DOHMH. Interviews were conducted with three categories of staff: leadership (i.e., buprenorphine program management or leadership, eight interviews), staff (i.e., buprenorphine coordinators or other staff, eleven interviews), and buprenorphine providers (six interviews). We identified themes related to barriers and facilitators to program implementation using thematic analysis. We make recommendations for implementation based on our findings.

**Results:**

Programs differed in their stage of development, location of services provided, and provider type, availability, and practices. Barriers to providing buprenorphine services at SSPs included gaps in staff knowledge and comfort communicating with participants about buprenorphine, difficulty hiring buprenorphine providers, managing tension between harm reduction and traditional OUD treatment philosophies, and financial constraints. Challenges also arose from serving a population with unmet psychosocial needs. Implementation facilitators included technical assistance from DOHMH, designated buprenorphine coordinators, offering other supportive services to participants, and telehealth to bridge gaps in provider availability. Key recommendations include: (1) health departments should provide support for SSPs in training staff, building health service infrastructure and developing policies and procedures, (2) SSPs should designate a buprenorphine coordinator and ensure regular training on buprenorphine for frontline staff, and (3) buprenorphine providers should be selected or supported to use a harm reduction approach to buprenorphine treatment.

**Conclusions:**

Despite encountering challenges, SSPs implemented buprenorphine services outside of conventional OUD treatment settings. Our findings have implications for health departments, SSPs, and other community organizations implementing buprenorphine services. Expansion of low-threshold buprenorphine services is a promising strategy to address the opioid overdose epidemic.

## Background

Opioid overdose deaths in New York City (NYC) more than doubled between 2010 and 2019, despite the availability of evidence-based opioid use disorder (OUD) treatment [1]. Buprenorphine is a safe and effective medication treatment for OUD that reduces non-prescribed opioid use [2], HIV risk behaviors [3], and opioid overdose mortality [4]. However, buprenorphine treatment is underutilized, in part because of barriers to treatment, such as provider availability or program practices that are burdensome for patients [5]. Low-threshold buprenorphine services seek to increase access to and acceptability of buprenorphine treatment for people with OUD. Low-threshold buprenorphine services are characterized by: (1) same-day treatment entry, (2) a harm-reduction orientation, (3) flexibility, and (4) availability in non-traditional settings [6]. A harm reduction orientation refers to non-judgmental provision of services and respecting patients’ goals, even if they do not intend to stop all drug use [7]. Increasing access to low-threshold buprenorphine services may help reduce opioid-related harms.

While low-threshold buprenorphine services can be provided in traditional medical settings [8], syringe services programs (SSPs) are ideally positioned to reach people with OUD who are at risk for overdose and marginalized from other sources of care [9, 10]. Historically, SSPs have increased access to other health services, such as STI testing, HIV and hepatitis C virus testing, and naloxone for opioid overdose reversal. In recent years, some SSPs in the USA have offered onsite buprenorphine services. Programs described in the literature have varied by location of services offered (e.g., mobile site, drop-in center) and treatment philosophy (e.g., requiring abstinence from illicit opioids, not conducting urine toxicology testing) [11–15]. Promising treatment outcomes have been reported, but to our knowledge, implementation of buprenorphine services in SSPs has not been studied systematically.

In NYC, 15 SSPs are currently funded by the NYC Department of Health and Mental Hygiene (DOHMH) to provide harm reduction services to approximately 16,000 participants per year. In 2017, DOHMH launched an initiative to support SSPs in developing low-threshold buprenorphine services as part of a multipronged initiative to reduce opioid-related overdoses. Our objectives were to provide the first qualitative evaluation of buprenorphine service implementation at SSPs. First, we categorized program characteristics. Then, we sought to identify barriers to and facilitators of implementing SSP-based buprenorphine services. We use these data to make recommendations for implementation of SSP-based buprenorphine services*.*

## Methods

### Program overview

In 2017, DOHMH funded nine SSPs to develop buprenorphine services. Funding generally covered staff time, including consultants and subcontractors, and program supplies and equipment. Awards to programs ranged from $87,000–218,000 yearly (USD). DOHMH additionally provided technical assistance to SSPs, which consisted of informational sessions on regulatory compliance (two-part training) and quarterly learning communities (five sessions) over 15 months (December 2018–February 2020). Program managers and at least one other staff member involved in buprenorphine services were required to attend compliance and learning community sessions. Topics covered are listed in Box 1.

**Box 1:** Topics covered during trainings on regulatory compliance and learning communities

Regulatory compliance	Electronic prescribing Health record maintenance Protection of health information Urine toxicology testing State and federal buprenorphine regulations
Learning communities	Buprenorphine (clinical information) Low-threshold principles Documentation of services Potential challenges Participant engagement and retention Urine toxicology testing

DOHMH also funded a harm reduction organization to conduct annual staff trainings to prepare staff to counsel SSP participants on treatment options. Programs were required to develop policies and procedures for buprenorphine services, for which DOHMH offered funding to hire consultants. Programs could request funding for an electronic health record (EHR) (software, installation fees, staff training, and a certain number of user licenses) in their budget proposals. Programs were encouraged to request individualized DOHMH technical assistance for any challenges they encountered in program development. Clinical mentoring was available from a harm reduction-experienced buprenorphine provider. All SSPs reported on buprenorphine services provided using an existing online reporting system developed by DOHMH for SSPs to report on other harm reduction services provided.

### Evaluation of implementation

This evaluation was deemed to not be human subjects research by the DOHMH Institutional Review Board. Two academic physicians (AJ and AF) unaffiliated with DOHMH evaluated SSPs’ experiences with implementing low-threshold buprenorphine services with support from DOHMH. AJ completed a total of 26 semi-structured qualitative interviews from April 2019—November 2019 at eight of the nine SSPs in NYC that received funding. One SSP was excluded because it developed buprenorphine services in collaboration with an academic medical center, so their experiences were not generalizable to the eight other SSPs. Interviews were conducted with three categories of staff: leadership (i.e., buprenorphine program management or leadership, eight interviews), staff (i.e., buprenorphine coordinators or other staff, eleven interviews), and buprenorphine providers (six interviews). In the staff category, all but one SSP staff member were employed by the SSP prior to development of buprenorphine services. SSP leadership selected staff and provider interviewees after researchers contacted them and explained the objectives of the study.

Semi-structured interviews followed a standard script that was developed in collaboration with DOHMH. Interview guides were tailored to the program role of the interviewee (Table 1). Twenty-two of the 26 interviews were conducted in-person, and four were conducted by phone. All interviews were audio-recorded, transcribed, and uploaded to Dedoose, a web-based tool for qualitative analysis. An analytic team comprised of two academic physicians (AJ and AF) and one qualitative researcher at DOHMH (AH) developed and iteratively refined a codebook consistent with study objectives. We used thematic analysis to first categorize program characteristics and second identify overarching themes related to barriers and facilitators to program implementation. Transcripts were then coded by one researcher (AJ).

**Table 1:** Interview topics by program role of interviewees

Program Role of Interviewees	Topics covered
Leadership	Barriers and facilitators to buprenorphine services development Staff training Strategies for participant engagement Identifying, hiring, and retaining buprenorphine providers Use of electronic health records Participant monitoring Regulatory compliance
Staff	Attitudes toward and knowledge of buprenorphine Buprenorphine treatment training received Strategies for participant engagement
Buprenorphine providers	Experience with buprenorphine treatment Experience with harm reduction Clinic workflows for buprenorphine prescribing Clinical decision making Perceptions of participants' challenges
All	Attitudes toward low-threshold buprenorphine services General facilitators and barriers to providing low-threshold buprenorphine services at SSPs

Findings from interviews are summarized describing prominent themes. We describe program characteristics without quotations for brevity. Data on barriers and facilitators of program implementation are supplemented with direct quotations from interviewees that provide context or highlight critical points. We provide recommendations for program implementation based on the findings from this study. Throughout the manuscript, we refer to people who use SSP services as “SSP participants”.

## Results

### Program characteristics

*Stage of buprenorphine development:* Of the eight SSPs included in the evaluation, five developed new buprenorphine programs and were active at time of interview, one SSP had a buprenorphine program established prior to DOHMH funding, and two SSPs initiated the development of new buprenorphine programs, but had to stop due to setbacks (one lost its office space; the other lost its provider). Characteristics of the six active buprenorphine programs are highlighted in Table 2 and additional details are provided below.

**Table 2:** Characteristics of low-threshold buprenorphine programs with active services at the time of the evaluation (*N* = 6)

		Program					
		A	B	C	D	E	F
Stage of development	Already running		X				
	New	X		X	X	X	X
Location	Organization's medical clinic			X			X
	New consultation area	X			X		
	Existing office space		X				
	Mobile unit					X	X
Availability	Limited hours	X	X			X	
	Full time services			X	X		
Provider	NP	X			X		
	PA			X	X		
	Physicians		X			X	X
SSP staff	Peer specialist	X	X			X	
	Buprenorphine coordinator	X	X	X	X	X	X
Documentation	Paper charts				X	X	
	Provider's own EHR	X					
	Medical clinic EHR			X			X
	SSP's data management software		X				
Urine toxicology testing	Existing infrastructure			X			X
	Participants sent to stand-alone laboratory					X	
	SSPs collect and send out	X	X		X		

### Buprenorphine providers

Buprenorphine providers were nurse practitioners, physician assistants, and physicians (family practice, psychiatry, and general internal medicine), employed part-time by the SSP or full-time by the organization’s medical clinic. One SSP partnered with an addiction medicine fellowship program to host a rotating addiction medicine provider-in-training. SSPs employed between one and three buprenorphine providers contracted for a set number of hours per week.

### Electronic documentation and prescribing

Programs varied in their use of electronic documentation. Organizations that had medical clinics used existing EHRs. At one program, the provider used their own cloud-based EHR which they also used in their private practice. Two programs used paper charts. One program used the SSP's existing data management software. All programs used electronic prescribing, in compliance with state and federal regulations.

### Buprenorphine coordinators

The six active programs all employed buprenorphine coordinators who were nurses, medical assistants, social workers, or other SSP staff with informal training in buprenorphine treatment**.** Buprenorphine coordinator roles included providing education and orientation to buprenorphine services; conducting eligibility screening; monitoring participant engagement; providing navigation services; coordinating with buprenorphine providers; supervising buprenorphine peer specialists and navigators; and SSP duties unrelated to buprenorphine treatment**.** Examples of navigation services included: making appointment reminder calls; contacting participants who were due for refills; helping with pharmacies and insurance authorizations; and providing psychosocial support (text messaging and phone calls to support participants in taking their medication and abstaining from non-prescribed opioids)**.**

### Participant engagement strategy

SSP staff promoted buprenorphine services using fliers, brochures, and conversations with existing participants at office and mobile sites. Three programs formally involved peers (i.e., SSP participants with lived experience of OUD) as buprenorphine champions or specialists to engage SSP participants who expressed interest in buprenorphine. These peers conducted community outreach at mobile sites and served as point-persons for other SSP staff members who identified SSP participants interested in buprenorphine. Interested participants were then connected with buprenorphine coordinators for more in-depth counseling and an introduction to buprenorphine services.

### Buprenorphine treatment policies and procedures

Buprenorphine can displace other opioids from opioid receptors and cause severe withdrawal symptoms if taken too soon, thus participants must wait until they are in moderate opioid withdrawal to take the first buprenorphine dose. Most programs used a “home induction” approach, where the provider instructed participants when and how to take the first dose of buprenorphine at home [16]. Some programs offered the option of “office-based induction” and one program required it, where participants would take the first buprenorphine dose at the SSP office, so a provider could monitor their level of withdrawal before and after starting buprenorphine. Three of the six active programs reported being able to consistently offer same-day treatment. The other programs did not, either because of lack of provider availability (one program), lengthy intakes during the first visit (one program), or requiring participants to be in withdrawal to receive a buprenorphine prescription (one program). Generally, participants were required to follow up with the provider weekly or every two weeks at the beginning of treatment and then were seen monthly after stabilizing. None of the programs required participants to participate in additional counseling beyond that which was routinely provided by providers.

### Urine toxicology tests

Programs performed urine toxicology testing at different frequencies, ranging from every buprenorphine visit to random intervals. Use of urine toxicology testing varied depending on the provider. No provider reported routinely stopping treatment for opioid-positive urine toxicology tests. Some providers increased the frequency of visits or spoke with participants about alternative treatments if they had multiple opioid-positive tests. All buprenorphine providers required that participants have buprenorphine-positive urine toxicology tests to continue treatment.

## Barriers to implementation

There were numerous barriers to providing buprenorphine services at SSPs. These included: staff knowledge and skills gaps, difficulty hiring and retaining buprenorphine providers, managing tension between harm reduction and traditional OUD treatment philosophies, and financial constraints. Challenges also arose from serving a population with unmet psychosocial needs.

### SSP leadership lacked experience implementing medical services

Program staff members reported that their leadership needed additional guidance at the beginning of implementation, particularly sites that did not have existing clinical infrastructure. Although leaders were experienced in managing nonprofits, many lacked experience building health service programs. Specifically, leaders lacked requisite knowledge regarding provider recruitment and contracting, malpractice insurance requirements, creating clinical policies and procedures, regulatory requirements, and electronic health records.

### Medical provider challenges

Provider-related challenges fell into two main categories: (1) *Hiring buprenorphine providers* and (2) *Comfort with harm reduction or “low-threshold” treatment principles.*

- (1) *Hiring buprenorphine providers:*

Programs found it challenging to identify buprenorphine providers who were experienced with buprenorphine treatment and willing to work part-time and in a harm reduction context. Covering malpractice insurance was prohibitively expensive for SSPs, and finding buprenorphine providers who had their own malpractice insurance was difficult, limiting the pool of potential candidates. Programs posted job listings online and asked personal or professional connections to advertise positions. Programs affiliated with medical clinics benefitted from established clinician recruiting teams. SSPs not affiliated with medical clinics hired buprenorphine providers for one to twelve hours per week, due to financial constraints, which was another challenge. However, finding the right provider was difficult: “We really don't have a provider that really understands the population.”—Program Coordinator (Program 2).

- 2) *Comfort with harm reduction or “low-threshold” treatment principles:*

Programs had difficulty finding harm reduction-oriented buprenorphine providers, and few buprenorphine providers had previous experience working in harm reduction settings:

“My approach has changed over time. Because when you study something at the beginning you, you're trying to kind of follow it to the letter –and I didn't have the concept of harm reduction either before I came. So I kind of learned as I have been here. —Buprenorphine provider (Program 4).

Program staff recognized this challenge:

“I think finding a provider that understands and truly practices harm reduction with their client is something that's rare. I feel like every provider has a tendency to be abstinence-based and use fear tactics when talking to their clients or their participants, finding someone that will implement [harm reduction] into their care has been a challenge.” —Program Manager (Program 3).

Some buprenorphine providers expressed concerns about their participant's continued opioid use. More than one provider were reluctant to provide buprenorphine prescriptions to participants who were also taking benzodiazepines. Individual buprenorphine providers had different practices around continuing to prescribe buprenorphine to participants who missed appointments. Buprenorphine providers also expressed concerns about their legal liability and risks to participants*:*

“I told you I'm a little bit of a control freak… And I'm like that with the buprenorphine because it is a controlled substance… And number one, I don't want to get myself in trouble… and number two, I also don't want to be so lackadaisical that someone else could hurt themselves… I'm responsible… I'm not giving it to you for you to hurt yourself.” —Buprenorphine provider (Program 4).

Harm reduction staff did not always agree with provider practices that conflicted with harm reduction principles or deviated from their understanding of clinical guidelines, but they were uncomfortable communicating this to buprenorphine providers: “It's a little difficult as to how we manage because we don't want to disrespect the doctor.” —Program Manager (Program 2).

Some staff members suggested that DOHMH should train buprenorphine providers in harm reduction principles, as they felt they had limited authority to give buprenorphine providers feedback on their practice. The state department of health offered a learning community for SSP buprenorphine providers, but attendance was voluntary, and buprenorphine providers often were unable to attend due to conflicting clinical schedules.

When programs were able to find a harm reduction-oriented provider, this was a major facilitator to program implementation. Harm reduction-oriented buprenorphine providers were able to engage with SSP participants and work effectively in non-traditional settings:

“And then we had [redacted], who’s a wonderful fit. She was with us I think for six months, she was really great. She was the one that was out in the mobile unit, was able to engage a lot of people into the program… She’s a harm reductionist, like she understood opioid use disorder in a way… that most prescribers that I’ve talked to have not understood it.” —Program Manager (Program 3).

### Differences between harm reduction and traditional OUD treatment philosophy

SSPs historically have not offered OUD treatment, and some programs noticed philosophical differences between traditional, abstinence-based treatment, and harm reduction approaches. Program leadership discussed concerns that offering buprenorphine (bupe) services would imply that the organization expected participants to stop non-prescribed opioid use.

What has been the biggest challenge with the program to date? (Interviewer)

“– well, it's about moving from not offering bupe into making it widely available without sending the message that you are being abstinence based.… But, when we talk to our clients (we) say this is an option… it all depends on how this relates to your life and to the things that you want to do.” —Leadership (Program 5).

Staff members also found it difficult navigating their roles as harm reductionists and helping people engage in buprenorphine services. One staff member spoke about how offering buprenorphine treatment changed their expectations for participants, leading to disappointment if participants resumed using non-prescribed opioids, which typically would be understood differently from a harm reduction perspective:

“I think it's sometimes, it's sometimes knowing that someone is going in the path that they want and all of a sudden, (they have) a big relapse. So that, emotionally for the harm reduction team as much as they want to keep the philosophy, it just really bothers the team… So that's a challenge, that it's hard to see, but because you're getting to the same level of the clients and you're not being pushy about it, but ask(ing) them what they want –– right, it gets a little bit more frustrating.” —Program Manager (Program 6).

### Staff knowledge and comfort communicating with participants about buprenorphine

Interviewees reported challenges with staff knowledge about buprenorphine at the beginning of program implementation. The annual staff training provided was perceived to be geared toward a medical audience, which was too technical for frontline staff. Even staff members closely involved in the program primarily learned about buprenorphine informally. For example, some staff members had personal experience with buprenorphine treatment, and others learned on the job from working closely with a provider or another buprenorphine program staff member. Programs also identified a need for refresher trainings for staff. Frontline staff desired training to help communicate quickly and effectively to SSP participants about buprenorphine:

“You know, with buprenorphine basically you have to understand that the clients don't really know too much. And basically the messaging [about buprenorphine] has to be really specific. It has to be something that might catch [participants’] attention.” —Leadership (Program 4).

### Participant-level challenges

SSP leadership and staff perceived participant challenges in the following categories: (1) *Unique characteristics*; (2) *Unmet service needs*; and (3) *Participants’ prior negative experiences with buprenorphine.*

(1) *Unique characteristics:* Some programs observed that unique characteristics of their participants tempered interest in buprenorphine services. One program served a young population, whom they perceived as lacking interest in OUD treatment. Another program was located near a methadone program, and most SSP participants were already enrolled in methadone treatment. Programs serving populations without stable housing noted the unique challenges of buprenorphine in this population:

“… they knew that buprenorphine was something that was mostly prescribed to specific populations meaning, you know, white America that were fully housed… and it was really, they didn't find it to really be like for them… so if I start this… I get a prescription where do I keep it, where do I store it, where do I put it. For clients who are chronically homeless that… certainly becomes a challenge…” —Leadership (Program 5).

To help address challenges with storage, some participants received small quantities of medication and returned multiple times per week for renewal of prescriptions. Other participants found pill boxing buprenorphine helpful to improve their adherence.

(2) *Unmet service needs*: A common theme was that buprenorphine alone did not meet all of participants’ needs. Staff perceived that participants required supportive services related to basic needs and buprenorphine to be successful in treatment. Services identified included peer navigations services, vocational training, and housing:

“We need other resources, viable resources that we can present to the clients for them to be adherent and stable in their life. I mean… some form of housing vouchers and even like some clothing, meals… employment, they just need realistic options – I think like right now, they don't really see a way forward… Okay, I'm getting this medication and stuff, I'm taking Suboxone, but these other things in my life ain't getting right… I think they need something to see in the future and I don't think they're really seeing it.” —Buprenorphine Coordinator (Program 4).

(3) *History of negative experiences with buprenorphine:* Many SSP participants reported having had past experiences of precipitated withdrawal when taking buprenorphine and were reluctant to try it again. In response, staff attempted to dispel misinformation about buprenorphine and counseled participants on how to avoid precipitated withdrawal when starting buprenorphine.

### Financial constraints

The primary financial constraint for programs was hiring buprenorphine providers. Most programs only had funding to hire a medical provider for a limited number of hours per week and could not afford buprenorphine providers without their own malpractice insurance coverage. Lack of funding also made it difficult to retain buprenorphine providers and mental health professionals in some organizations. SSPs not associated with medical clinics reported that electronic health records were unaffordable. SSPs not affiliated with a medical clinic were also unable to bill insurance for medical services and thus relied exclusively on grant funding to sustain the program.

“…The salary ranges have been a pretty big issue…[employees] could also leave here and make $10,000 more somewhere else… Buying supplies can somewhat be a challenge… making sure we have enough MetroCards for our clients. Because we give them train fare for all their medical appointments… I think if we can give clients incentives – not for their medical appointment, but for like meeting with my staff that would help engage them into care, but we can't afford – incentives right now.” —Program Manager (Program 3).

Sustainability was particularly difficult for programs operating out of mobile vehicles. Upkeep and cost of repairing mobile vehicles was a barrier for sustainability. Finding buprenorphine providers who were interested and skilled in working in a mobile setting was also challenging.

## Facilitators to implementation

### Provider model

SSPs that were part of organizations with medical clinics had the greatest capacity to provide regular services and same-day buprenorphine treatment. Other programs were able to successfully contract with part-time buprenorphine providers when these buprenorphine providers had their own malpractice insurance (either independently or through another organization) and were willing to extend their availability via telehealth. Provision of remote services via telehealth helped bridge gaps when in-person hours were unavailable. Two programs arranged for telephonic follow-up if participants came to the SSP when the provider was not available in-person. One program compensated their provider (using grant funding) for an additional 1.5 hours per week for telehealth visits to attend to participants who had been unable to attend in-person appointments and facilitate prescription refills. Buprenorphine coordinators were crucial to maintaining continuity of care in programs with limited provider hours.

### Technical assistance from DOHMH

Technical assistance from the DOHMH was the key in several areas, particularly in developing policies and procedures*.*

At the beginning of the initiative, as part of the funding requirements, programs were asked to create policies and procedures which included protocols for starting buprenorphine, follow-up intervals for participants, and laboratory testing tailored for their organization and participants. However, many programs struggled, having little experience creating clinical protocols. Two SSPs hired consultants using funds provided by DOHMH, but other programs could not identify consultants with the necessary expertise. DOHMH later provided templates and individualized assistance to SSPs to develop their own policies and procedures, a key facilitator to programs that did not hire consultants.

The DOHMH also assigned a single staff member as the point person to answer questions that arose during the implementation process. This point person assisted programs with a range of challenges, including finding buprenorphine providers and addressing medicolegal concerns (legal liability associated with providing clinical services).

“And [DOHMH staff member] was very helpful and responsive… it was helpful to have conversations because we would identify and then look at issues that hadn't been thought of in advance… I was focused to some extent on… risk management for the organization, right; making sure that we were not going to have the state health department… breathing down our necks because we were providing services in some way, that, you know, was considered too broad… And then there were questions around, you know, insurance and whose insurance covered what, if it was under the individual, their malpractice. Policy and procedures, questions, data questions…” —Leadership (Program 1).

### Dedicated buprenorphine coordinators

DOHMH encouraged SSPs to designate a dedicated buprenorphine coordinator. Programs who followed this advice reported that it was a facilitator of program success. Buprenorphine coordinators gained participants’ trust, perhaps more easily than buprenorphine providers:

“So for the most part, our clients are kind of honest in telling us things… And I told them like if you're using, you know, I ain't going to stop you from getting prescribed… I mean, that's not what I'm here for.… How could I help you maintain your adherence to Suboxone and stop you from using—and some like just need to talk.” —Buprenorphine Coordinator (Program 3).

At one program, coordinators collaborated closely with buprenorphine providers to identify ways to support participants:

“…Me and the providers got together and we started identifying clients that were high risk of failure or at risk of failure, for whatever variety of reasons, and then we'll come in, collaborate with the doctor and the client at the same time – and work out a plan as to okay, this is how we can work this client through this part of his life to become stable on Suboxone.” —Buprenorphine Coordinator (Program 3).

### Robust participant support

Several SSPs offered more support services than typically can be provided in a doctor’s office. Buprenorphine coordinators and peers provided a variety of navigation and support services. At one SSP, peer navigators (supervised by the buprenorphine coordinator) accompanied participants to healthcare appointments, conducted home visits, and, when needed, delivered MetroCards the day before appointments. The following quote details some of the auxiliary supports offered at another SSP:

“…And we do everything possible, calling insurances, walking to the pharmacy – so like every step of the way – we make sure you get [buprenorphine] and nothing happens in between from the van to the pharmacy… We have that urgency like you're here now, we're getting this for you now.” —Buprenorphine Coordinator (Program 2).

### Relationship with pharmacy

Establishing a relationship with a local pharmacy able to stock and dispense buprenorphine was a key facilitator for four programs. Staff could be confident that buprenorphine would be in stock (including a variety of strengths and formulations, depending on what participants’ insurance covered), participants would be treated respectfully, and pharmacies would help troubleshoot insurance problems. Pharmacies affiliated with federally qualified health centers were able to provide discounted medication through the 340B Drug Pricing program [17]. One pharmacy delivered prescriptions directly to the SSP for onsite buprenorphine initiation.

“Well, I've been fortunate that the pharmacy we deal with is actually pretty good and – with the population that we serve, you know, there's always that that, that stigma… and they have been looked at differently, not him (pharmacist)… He greets them, he speaks to them like folks.” —Buprenorphine Coordinator (Program 6).

### Recommendations for implementation of SSP buprenorphine services

Taken in total, these interviews provide key lessons learned for implementing low-threshold buprenorphine services at SSPs. Below we summarize our recommendations for key stakeholders (health departments, SSPs, and buprenorphine providers) based on the barriers and facilitators we identified in this report (Table 3).

**Table 3:** Recommendations for stakeholders in SSP buprenorphine services implementation

**Health departments**
Provide robust support for: 1) Building clinical infrastructure (e.g., health record, billing systems) 2) Developing policies and procedures 3) Addressing medicolegal concerns (e.g., malpractice insurance, legal liability associated with providing clinical services) 4) Selecting and training buprenorphine providers in harm reduction principles 5) Training frontline SSP staff to counsel participants about buprenorphine Designate a point-person who can provide individualized technical assistance to SSPs
**SSPs**
Train buprenorphine providers in harm reduction principles and facilitate a system for staff to safely provide feedback on practices
Ensure regular training on buprenorphine for SSP staff
Compensate buprenorphine providers for telehealth visits
Elicit SSP participant input on buprenorphine program design
Develop and update buprenorphine services policies and procedures through collaborative discussion with staff, buprenorphine providers and participants
Designate a dedicated buprenorphine services coordinator
Establish a relationship with a local pharmacy
Link participants to supportive services as needed (housing, mental health services, vocational training, etc.)
**Buprenorphine providers**
Past experience or dedicated time for training in: 1) Low-threshold treatment principles and practices 2) Harm reduction principles and practices
Work collaboratively with harm reduction staff, particularly: 1) Soliciting and incorporating feedback from team members 2) Identifying and addressing client goals and basic needs

## Discussion

This study aimed to identify barriers to and facilitators of implementation of SSP-based low-threshold buprenorphine services and make recommendations for implementation. We found that most programs successfully implemented at least some buprenorphine services despite experiencing challenges related to the novelty of providing buprenorphine services onsite and finding buprenorphine providers. Programs with pre-existing clinical infrastructure had many advantages in implementing and sustaining buprenorphine services. Many SSPs throughout the USA do not have this advantage and would benefit from support from public health agencies for developing clinical infrastructure, selecting and training providers, and training staff. Overall, SSPs are promising sites to expand access to low-threshold buprenorphine services.

At SSPs, buprenorphine providers are generally not onsite full-time, therefore, having dedicated staff who can provide continuity is crucial. As such, we recommend having a dedicated buprenorphine coordinator to facilitate program implementation and ongoing management. This has been demonstrated in HIV treatment settings and is a key component of the “Massachusetts Model” of office-based buprenorphine treatment [18, 19]. Similarly, other programs in low-threshold settings have used nurse care managers, in which nurses play central roles in completing initial assessments, counseling participants about initiation procedures, conducting follow-up visits, obtaining and discussing urine toxicology results, and discussing dose changes [20, 21]. Maximizing collaboration between buprenorphine providers and other SSP staff members is particularly important for low-threshold settings.

Other program characteristics that differed between sites may also facilitate implementation. SSPs were able develop successful programs within drop-in centers, mobile units or in partnership with established community health centers. However, not all programs were able to hire a harm-reduction oriented provider, which was an essential component of successful programs. Programs also differed in their involvement of peers. Few programs formally involved peers in buprenorphine services. When they were formally involved, peers served as participant navigators, provided other supportive services, and played critical roles in engaging participants. Training peers in buprenorphine and involving them in implementation of buprenorphine services could be an important strategy to improve the reach of buprenorphine services.

Establishing successful SSP-based buprenorphine services will also require confronting philosophical differences between OUD treatment and harm reduction. Heller and colleagues described these differences in reference to implementing HIV care at SSPs, highlighting that traditional medical models are hierarchical, center around physician expertise, and expect patients to be compliant with prescribed treatment plans [22]. Our finding that harm reduction staff expressed discomfort in providing feedback to buprenorphine providers may reflect this hierarchy. Harm reduction models emphasize inclusivity, collaborative decision-making, and valuing small changes. Medical practice has begun to embrace more patient-centered approaches [23], but as exemplified by the provider who commented, “I'm not giving it to you for you to hurt yourself,” some clinicians may view their role in making prescribing decisions less collaboratively. Specific to buprenorphine treatment, accepting patient-centered treatment goals, including managing and reducing opioid use as opposed to stopping non-prescribed opioid use completely, could lead to better collaboration. Accordingly, buprenorphine providers can and should be trained in harm reduction principles [8]. Giving buprenorphine providers clear guidance about what prescribing practices are allowable could assuage concerns about legal liability. For example, buprenorphine providers expressed concerns about prescribing buprenorphine to SSP participants who took benzodiazepines; however, in 2017, the US Food and Drug Administration provided guidance that withholding buprenorphine from patients who use benzodiazepines or other sedatives could increase risk due to untreated OUD [24]. Changing medical culture to embrace harm reduction will require training and feedback, both of which could be provided by provider champions who are trusted messengers [25]. Infusing traditional OUD treatment with harm reduction principles could both boost program engagement and protect participant safety.

Implementing buprenorphine services at SSPs also requires additional attention to financial sustainability. Programs were funded by a large city health department as part of a major multi-sector strategy to reduce overdose deaths. Significant financial support is needed to hire buprenorphine providers and pay for malpractice insurance. Innovations in the malpractice market are necessary to make contracting with individual buprenorphine providers more feasible for SSPs. Until then, in places where there are multiple SSPs or community organizations that wish to implement buprenorphine services, organizations may be able to partner with a medical clinic and provide funding for them to lend a part-time buprenorphine provider. Alternatively, health departments could employ buprenorphine providers to work in SSPs. In some states, SSPs may be able to bill for medical services, including buprenorphine services. Health departments and SSPs should explore whether SSPs billing for buprenorphine services would create a viable revenue stream and increase program sustainability or whether the start-up costs and staffing costs for billing would be too high. In some states, policy changes may be required to allow SSPs to bill for health services. Buprenorphine treatment is highly cost effective to society due to reductions in patients’ use of emergency health services and criminal-legal involvement [26]. Thus, adequately funding programs could be a wise investment for communities with high levels of opioid-related harms.

### Strengths of this study

Interviewing individuals from multiple programs at different stages of development provided a diversity of models and perspectives on barriers and facilitators throughout the implementation process. Interviews were conducted with individuals with varying roles at the SSPs, including buprenorphine providers, leadership, buprenorphine coordinators, and other SSP staff. Finally, members of the study team were from outside DOHMH, reducing some potential biases in the study.

### Limitations

We used strictly qualitative methods, so data were not collected systematically on process measures such as number of staff trainings SSPs held or number of SSP participants approached about buprenorphine treatment. The study was conducted up to two years after implementation, introducing recall bias and reducing the opportunity to act on program feedback in a timely manner. The study interviewed SSP staff but not participants, so may have missed important perspectives of those most impacted. Finally, we only interviewed SSP stakeholders, not DOHMH staff, so the perspective of the funders was not formally examined.

### Future directions

Our finding that offering buprenorphine services may change SSP participants’ and staff members’ perceptions of the SSP’s harm reduction mission deserves additional investigation. While we did not examine participant perspectives in this study, some SSPs reported concern that participants would question the organization’s commitment to harm reduction after they started offering buprenorphine services. Staff members also reported shifts in their expectations for participants who engaged in treatment, expressing hopes for consistent adherence to buprenorphine, abstinence from non-prescribed opioids, and greater stability in participants’ lives. Some staff were concerned that such change in expectation would compromise their non-judgmental stance toward a participant’s substance use. It is important to support staff and organizations in exploring their understanding and practice of harm reduction and an evolving understanding of harm reduction principles and OUD treatment applied in new contexts. Lastly, this study examined implementation of low-threshold buprenorphine services, but understanding SSP participants’ experiences with such services will be an important area of future study.

## Conclusions

Despite encountering challenges, eights SSPs in NYC have implemented buprenorphine services with DOHMH support, serving a population at risk for opioid-related harms that may be reluctant to seek treatment elsewhere. Lessons learned from this study can be used to support SSPs and other community organizations in developing and improving buprenorphine services. Over time, SSPs have adapted to community needs in providing sterile syringes, distributing naloxone, and now improving access to lifesaving OUD treatment. SSPs are valuable community resources that improve the health of people who use drugs.

## Data Availability

Not applicable. This study only used qualitative data.

## References

[1] New York City Department of Health and Mental Hygiene. Unintentional Drug Poisoning (Overdose) Deaths in New York City in 2019. Epi Data Br [Internet]. 2020;122. Available from: http://www1.nyc.gov/assets/doh/downloads/pdf/epi/datatable66.pdf

[2] Mattick RP, Breen C, Kimber J, Davoli M (2014). Buprenorphine maintenance versus placebo or methadone maintenance for opioid dependence. Cochrane Database Syst Rev.

[3] Edelman EJ, Chantarat T, Caffrey S, Chaudhry A, O’Connor PG, Weiss L (2014). The impact of buprenorphine/naloxone treatment on HIV risk behaviors among HIV-infected, opioid-dependent patients. Drug Alcohol Depend.

[4] Sordo L, Barrio G, Bravo MJ, Iciar Indave B, Degenhardt L, Wiessing L, Ferri M, Pastor-Barriuso R (2017). Mortality risk during and after opioid substitution treatment: systematic review and meta-analysis of cohort studies. BMJ.

[5] Gryczynski J, Mitchell SG, Jaffe JH, O’Grady KE, Olsen YK, Schwartz RP (2014). Leaving buprenorphine treatment: patients’ reasons for cessation of care. J Subst Abuse Treat.

[6] Jakubowski A, Fox A (2020). Defining low-threshold buprenorphine treatment. J Addict Med.

[7] National Harm Reduction Coalition. Principles of Harm Reduction [Internet]. 2020 [cited 2021 Apr 6]. Available from: https://harmreduction.org/about-us/principles-of-harm-reduction/

[8] Kapadia SN, Griffin JL, Waldman J, Ziebarth NR, Schackman BR, Behrends CN (2021). A harm reduction approach to treating opioid use disorder in an independent primary care practice: a qualitative study. J Gen Intern Med.

[9] Pizzicato LN, Johnson CC, Viner KM (2019). Correlates of experiencing and witnessing non-fatal opioid overdoses among individuals accessing harm reduction services in Philadelphia, Pennsylvania. Subst Abuse.

[10] van Boekel LC, Brouwers EPM, van Weeghel J, Garretsen HFL (2013). Stigma among health professionals towards patients with substance use disorders and its consequences for healthcare delivery: systematic review. Drug Alcohol Depend.

[11] Bachhuber MA, Thompson C, Prybylowski A, Benitez J, Mazzella S, Barclay D (2018). Description and outcomes of a buprenorphine maintenance treatment program integrated within prevention point Philadelphia, an urban syringe exchange program. Subst Abuse.

[12] Carter J, Zevin B, Lum PJ (2019). Low barrier buprenorphine treatment for persons experiencing homelessness and injecting heroin in San Francisco. Addict Sci Clin Pract.

[13] Hood JE, Banta-Green CJ, Duchin JS, Breuner J, Dell W, Finegood B (2019). Engaging an unstably housed population with low-barrier buprenorphine treatment at a syringe services program: lessons learned from Seattle, Washington. Subst Abus.

[14] Stancliff S, Joseph H, Fong C, Furst T, Comer SD, Roux P (2012). Opioid maintenance treatment as a harm reduction tool for opioid-dependent individuals in New York City: the need to expand access to buprenorphine/naloxone in marginalized populations. J Addict Dis.

[15] Hill K, Nussdorf L, Mount JD, Silk R, Gross C, Sternberg D, Bijole P, Jones M, Kier R, Mccullough D, Mathur P, Kottilil S, Masur H, Kattakuzhy Sarah, Rosenthal ES (2022). Initiation of low-threshold buprenorphine in nontreatment seeking patients with opioid use disorder engaged in hepatitis C treatment. J Addict Med.

[16] Cunningham CO, Giovanniello A, Li X, Kunins HV, Roose RJ, Sohler NL (2011). A comparison of buprenorphine induction strategies: Patient-centered home-based inductions versus standard-of-care office-based inductions. J Subst Abuse Treat.

[17] Health Resources and Service Administration. 340B Drug Pricing Program | Official web site of the U.S. Health Resources Services Administration [Internet]. HRSA. 2021 [cited 2021 Apr 12]. Available from: https://www.hrsa.gov/opa/index.html

[18] Weiss L, Netherland J, Egan JE, Flanigan TP, Fiellin DA, Finkelstein R (2011). Integration of buprenorphine/naloxone treatment into HIV clinical care: Lessons from the BHIVES collaborative. J Acquir Immune Defic Syndr.

[19] Korthuis PT, McCarty D, Weimer M, Bougatsos C, Blazina I, Zakher B, Grusing S, Devine B, Chou R (2016). Primary care–based models for the treatment of opioid use disorder: a scoping review. Annals Int Med.

[20] Hood JE, Banta-Green CJ, Duchin JS, Breuner J, Dell W, Finegood B, Glick SN, Hamblin M, Holcomb S, Mosse D, Oliphant-Wells T, Shim MHM (2020). Engaging an unstably housed population with low-barrier buprenorphine treatment at a syringe services program: lessons learned from seattle, Washington. Subst Abuse.

[21] Fine DR, Lewis E, Weinstock K, Wright J, Gaeta JM, Baggett TP (2021). Office-based addiction treatment retention and mortality among people experiencing homelessness. JAMA Network Open.

[22] Heller D, McCoy K, Cunningham C (2004). An invisible barrier to integrating HIV primary care with harm reduction services: philosophical clashes between the harm reduction and medical models. Public Health Rep.

[23] Epstein RM, Street RL (2011). The values and value of patient-centered care. Ann Fam Med.

[24] Center for Drug Evaluation and Research. Drug Safety and Availability - FDA Drug Safety Communication: FDA urges caution about withholding opioid addiction medications from patients taking benzodiazepines or CNS depressants: careful medication management can reduce risks. FDA.gov. 2017. 2: 1–6.

[25] Chauhan BF, Jeyaraman M, Mann AS, Lys J, Skidmore B, Sibley KM (2017). Behavior change interventions and policies influencing primary healthcare professionals’ practice-an overview of reviews. Implement Sci.

[26] Murphy SM, Jeng PJ, Poole SA, Jalali A, Vocci FJ, Gordon MS (2020). Health and economic outcomes of treatment with extended-release naltrexone among pre-release prisoners with opioid use disorder (HOPPER): protocol for an evaluation of two randomized effectiveness trials. Addict Sci Clin Pract.

